# Angiostrongylosis in Animals and Humans in Europe

**DOI:** 10.3390/pathogens10101236

**Published:** 2021-09-25

**Authors:** Eric R. Morgan, David Modry, Claudia Paredes-Esquivel, Pilar Foronda, Donato Traversa

**Affiliations:** 1 School of Biological Sciences, Queen’s University Belfast, Belfast BT9 5DL, UK; 2 Biology Center, Institute of Parasitology, Czech Academy of Sciences, 37005 Ceske Budejovice, Czech Republic; modryd@vfu.cz; 3 Department of Veterinary Sciences, Faculty of Agrobiology, Food and Natural Resources/CINeZ, Czech University of Life Sciences Prague, 16500 Praha-Suchdol, Czech Republic; 4 Department of Botany and Zoology, Faculty of Science, Masaryk University, 61137 Brno, Czech Republic; 5 Applied Zoology and Animal Conservation Group, University of the Balearic Islands, 07122 Palma de Mallorca, Spain; claudia.paredes@uib.es; 6 University Institute of Tropical Diseases and Public Health, University La Laguna, Tenerife, Canary Islands, 38200 San Cristóbal de La Laguna, Spain; pforonda@ull.edu.es; 7 Faculty of Veterinary Medicine, University of Teramo, 64100 Teramo, Italy; dtraversa@unite.it

**Keywords:** *Angiostrongylus*, angiostrongyliasis, lungworm, epidemiology, climate change

## Abstract

Lungworms in the genus *Angiostrongylus* cause disease in animals and humans. The spread of *Angiostrongylus vasorum* within Europe and the recent establishment of *Angiostrongylus cantonensis* increase the relevance of these species to veterinary and medical practitioners, and to researchers in parasitology, epidemiology, veterinary science and ecology. This review introduces the key members of the genus present in Europe and their impacts on health, and updates the current epidemiological situation. Expansion of *A. vasorum* from localized pockets to wide distribution across the continent has been confirmed by a rising prevalence in foxes and increasing reports of infection and disease in dogs, while the list of carnivore and mustelid definitive hosts continues to grow. The tropically distributed rat lungworm *A. cantonensis*, meanwhile, has been recorded on islands south of Europe, previously the Canary Islands, and now also the Balearic Islands, although so far with limited evidence of zoonotic disease. Other members of the genus, namely, *A. chabaudi*, *A. daskalovi* and *A. dujardini*, are native to Europe and mainly infect wildlife, with unknown consequences for populations, although spill-over can occur into domestic animals and those in zoological collections. The epidemiology of angiostrongylosis is complex, and further research is needed on parasite maintenance in sylvatic hosts, and on the roles of ecology, behaviour and genetics in disease emergence. Improved surveillance in animals and humans is also required to support risk assessments and management.

## 1. Introduction

The genus *Angiostrongylus* sits within the nematode superfamily Metastrongyloidea, alongside other lungworms that infect a wide range of mammalian species [1]. Around 20 species have been identified in the genus, although they have been subject to repeated taxonomic revision. The geographic and host distribution of the different species varies widely, as do their implications for animal and human health. This review includes only species currently present in Europe and takes a local perspective on their epidemiology.

*Angiostrongylus* species can be divided broadly into two groups: those using carnivores as definitive hosts (DHs), and those using rodents. In both cases, gastropod mollusc (snail and slug) intermediate hosts (IHs) are necessary for completion of the life cycle, and in comparison to mollusc-borne trematodes, the range of suitable IH species is extremely broad. Additionally, paratenic hosts can be involved in the life cycle, and some vertebrates can act as IHs. Animals can also be accidental, dead-end hosts for the parasite, but nevertheless suffer disease; in the case of humans, this causes zoonotic angiostrongyliasis [2,3,4]. The wide variety of host species involved in transmission and disease underpins complex epidemiology, which is only partly understood and likely to differ according to local or regional contexts.

In Europe, the *Angiostrongylus* species receiving most attention historically has been *Angiostrongylus vasorum*, because it is common, expanding, and a recognized cause of severe disease in domestic dogs [5]. Increasing research interest in *A. vasorum* has produced new tools for the diagnosis and treatment of infected dogs, as well as new discoveries around its host range, biology and epidemiology [6]. Prevalence in foxes can be high and mustelids appear to be suitable hosts, although the ecology and impacts of this species in wildlife hosts remain largely unstudied. Other *Angiostrongylus* spp. in carnivores and mustelids in Europe are *Angiostrongylus chabaudi* in cats [7] and *Angiostrongylus daskalovi* in badgers [8]. Similarly to *A. vasorum*, these exist in sylvatic cycles of unknown significance for host populations, whereas *A. chabaudi* can occasionally spill over to cause disease in domestic cats.

The second group of *Angiostrongylus* spp., infecting rodents as definitive hosts, has largely been ignored in Europe as being absent or of little significance. This has changed markedly with the arrival of *Angiostrongylus cantonensis*. Although this species was previously recognized on the Canary Islands, which are territorially European but distant from the continent [9], its detection on the Balearic Islands [10] makes it indisputably present in Europe. This is cause for concern, given that *A. cantonensis*-induced eosinophilic meningitis (AEM) in people in tropical and sub-tropical regions of the world is a disease which is difficult to prevent, diagnose and treat, and can be fatal [2,4]. A second rodent-borne species, *Angiostrongylus dujardini*, is native to Europe and comes to attention when infecting accidental hosts in zoos, causing disease in meerkats and primates [11].

Range expansion within Europe is so far evident only for *A. vasorum*; however, the prospect of future spread is an important consideration across species within the genus, along with the likelihood of consequent increases in disease. Within parasite ranges, ecological, behavioural and genetic factors might influence the extent to which exposure occurs and disease arises in definitive and accidental hosts. This review aims to provide an update on the current epidemiological situation of *Angiostrongylus* spp. in Europe, with essential information on the life cycle and pathogenesis of the different species, and then to address the important knowledge gaps that limit predictions around future disease risks.

## 2. *Angiostrongylus vasorum* (Baillet, 1866)

### 2.1. Life Cycle and Pathogenesis

The life cycle, as for other *Angiostrongylus* spp., is indirect [12]. Dogs and foxes are the main DHs, although natural infections have also been observed in other wild carnivores including wolfs, jackals, coyotes, raccoon dogs, stoats, weasels, and invasive American minks [13,14]. Adult worms reside in the right side of the heart and pulmonary arteries, and females produce eggs that are carried to the capillaries and hatch to first-stage (L1) larvae. After penetrating into the alveoli, the L1 larvae are carried by the broncho-ciliary escalator to the pharynx, swallowed, and pass out in the faeces [13]. There, they are ingested by gastropod molluscs, i.e., snails and slugs, which are the main IHs, although frogs can also fill this role [15]. Development continues in the IH to the third larval stage (L3), which is infective to the DH when ingested.

A wide range of IH species have been recorded with natural or experimental infections. Virtually every species of gastropod in which experimental infection has been attempted proved to be capable IH; surveys of natural gastropod populations in Europe have found infections in a long and growing list of native species [16,17,18,19,20]. Paratenesis has been demonstrated, in that L3 larvae penetrate into the tissues of frogs [15] and chickens [21] that are fed infected gastropods, and remain infective to DHs. Liberation of L3 larvae has also been demonstrated from gastropods, after which larvae survive outside the IH for several days [22,23].

Infection in dogs most commonly causes respiratory disease, which is associated with inflammation around L1 larvae as they penetrate the alveoli, i.e., verminous pneumonia [6]. Disease presents as coughing and dyspnoea and can be severe, although other outcomes include chronic fatigue, anorexia and syncope [24]. In a minority of cases, coagulation functions are impaired, and this provokes highly variable clinical outcomes depending on the site of the bleeding, which can be into the gut, lungs, abdomen or other organs including the brain or spinal cord [5,6]. Unusual clinical cases can also arise from larval migration, especially in kidneys and heart muscle [25], and even adult development, for example, in the eye [26].

Disease in other DHs is similar, i.e., verminous pneumonia with instances of larval migration in various organs. In foxes, lung damage in natural and experimental infections can be severe, and right-sided myocardial thickening [27] indicates elevated pulmonary arterial pressure. It seems likely that fitness is affected in heavily infected wild individuals, but the population-level consequences of parasitism are unknown. Infection with *A. vasorum* has also been recorded in a cat, which developed verminous pneumonia, but the infection did not become patent [28]. Patent infections in animals in zoological collections in Europe have been associated with primarily respiratory disease, notably in red pandas and meerkats [29].

### 2.2. Epidemiology

#### 2.2.1. Spread within Europe

The history of *A. vasorum* in Europe is characterized by a highly localized known distribution for the first 150 years after its discovery, and then apparently explosive spread across the continent. Until the 1990s, ‘hotspots’ of infection in dogs and foxes were recognized in small parts of the United Kingdom (UK) and Denmark, with records also locally from France, Italy and Spain [13,30]. Since then, increasing distribution has been recorded in both dogs and foxes: for example, in the United Kingdom, there was northward spread in fox populations [31] and autochthonous cases in dogs distant from previously known endemic areas [16]; similarly, spread was recorded in Switzerland [32] and Denmark [14]; and in Italy, in both foxes and wolves [33,34]. Increasing awareness has probably contributed to this apparent spread, especially since the development of high-throughput diagnostic tests suitable for large prevalence surveys. Immuno-diagnosis of parasite antigen and specific antibodies, for example, have led to new records in a large number of European countries in the past 10 years, with prevalence consistently between 0.5% and 3% in canine populations e.g., [35,36,37]. This perhaps indicates the discovery of areas of long-standing parasite presence, and the illusion of geographic spread due to increased detection. In areas with longitudinal data from foxes, however, spectacular rises in prevalence have been recorded, for example, a doubling over 8 years in the UK [31] and from around 20% to 80% over five years in Switzerland [32]. It is, therefore, highly likely that this species has actually increased in distribution and abundance in Europe.

#### 2.2.2. Risk Factors for Disease

Infection in dogs is associated with young age, autumn/winter season and lack of recent prior administration of anthelmintic treatment, although other factors such as breed are less clear [13,24]. Presumably, dogs infected with higher worm burdens and over a longer period are more likely to present with disease, although this has not been explicitly determined, and other factors such as host genetics might modify pathogenesis. The mechanisms of bleeding disorders observed in dogs infected with *A. vasorum*, in particular, are poorly understood [5,6].

Based on the life cycle, opportunities for contact with infected gastropod IH are likely to increase the risk of infection and disease. Intentional ingestion by dogs of gastropods and paratenic hosts such as frogs is possible, although unintentional consumption via contaminated food, or through behaviour such as scavenging or grass-chewing, are assumed to be more common. Food-borne transmission in dogs is possible not only by gastropod contamination, but also via paratenic hosts such as chickens [21], and although the cooking or freezing of meat would presumably reduce or eliminate larval viability, this has not been investigated. L3 larvae liberated from gastropods into water are infective [23]; therefore, dogs and other DHs could also be infected by ingesting water or food contaminated with free L3 larvae, although this has not yet been demonstrated in natural conditions. The range of paratenic host species in nature is almost certainly much broader than that documented so far [15,21], and trophic relationships between IHs, paratenic hosts and wild carnivore DHs no doubt underpin the maintenance of *A. vasorum* sylvatic cycles.

As understanding of the *A. vasorum* life cycle and ecology develops, dog owners might be presented with better-evidenced choices for avoiding infection of their pets. Similar principles might be applied in zoological collections. Given the current state of knowledge, however, the mainstay of disease control in pets is the regular administration of a suitable anthelmintic, in order to prevent the build-up of infection and consequent pathology [5,6].

#### 2.2.3. Prospects for Further Spread

The factors underlying emergence of *A. vasorum* in Europe are debatable: the urbanization of foxes, invasion of exotic snail species, and climate change have all been proposed, but evidence is inconclusive. Different factors appear more or less plausible in different areas (Table 1). It could be that all factors apply, but to different extents regionally, and that epidemiology and emergence are highly context-dependent. Globally, historical distribution has also been focal, but with evidence of recent spread, for example, in North America [38,39]. In almost all cases, observed expansion has been within areas noted to be climatically suitable, based on a combination of plausible thermal tolerance and observed discontinuities in historical distribution in foxes [40]. Consequently, climate seems to be important to *A. vasorum* distribution, but climate change cannot entirely explain the geographic spread over recent decades.

Ambiguity in the links between *A. vasorum* spread and climatic and other factors makes it difficult to evaluate the prospects of further range expansion, and indeed, changes in prevalence and disease impact within the existing range. Climate warming is most likely to accelerate larval development within poikilothermic gastropod IHs [40], but it remains uncertain as to whether this will translate into the increased availability of infective larvae to DHs. Temperatures experienced by developing larvae could exceed optimal development ranges in some areas and be modified by IH behaviour, whereas effects of altered rainfall patterns could be complex because they act on free-living larval stages as well as gastropod populations and availability to DHs. Within Europe, the range of *A. vasorum* is already broad, suggesting that expansion into suitable habitats is well-advanced, and research attention might usefully turn to aspects of environment and host ecology that promote high prevalence and spill-over into domestic and captive carnivore populations.

## 3. *Angiostrongylus chabaudi* (Biocca, 1957)

### 3.1. Life Cycle and Pathogenesis

To date, patent angiostrongylosis (i.e., shedding of L1 larvae) has been described only in the European wildcat (*Felis silvestris*), which is the natural host of *A. chabaudi* in Southern and Eastern Europe [47,48,49,50]. A few domestic cats from Italy have been found harbouring only immature/unfertilized *A. chabaudi* (reviewed in [50]).

In accordance with the biology of the *Angiostrongylus* genus, it is plausible that felids become infected by ingesting infective larvae (L3) in IH. The role of paratenic hosts is unknown but cannot be excluded. Molluscs were predicted to be intermediate hosts of *A. chabaudi* and, as expected, an experimental study showed that this species reaches the L3 stage in the land snail *Cornu aspersum* [51]. Other wild-caught gastropods (e.g., *Helix lucorum*, *Massylaea vermiculata*, *Lymax comemenosi*) have been recently found to harbour larvae of *A. chabaudi* in an endemic area of Greece [52]. Conversely, *A. chabaudi* has not been detected in any of the gastropods collected in other surveys in South America (reviewed in [50]) or in European countries bordering Italy, i.e., Austria [19].

While data on the effect of temperature and hibernation on the larval development in molluscs have been recently produced for other related metastrongyloid ‘lungworms’, notably *Troglostrongylus* and *Aelurostrongylus* [52,53], no similar information is available on drivers which may affect the development of *A. chabaudi* in gastropod intermediate hosts. Analogously, knowledge on its life cycle in felid hosts after infection is nil [50].

Practically nothing is known on the impact of *A. chabaudi* on the health and welfare of domestic cats and data available come exclusively from histopathological examinations of wild felids. To date, *A. chabaudi* is not considered a concern for domestic cats nor it is involved in the differential diagnosis of cardiorespiratory diseases [7,54]. Studies are advocated to understand if infections by immature stages may cause pathology in the lung arteries and parenchyma of domestic cats.

The most frequent lesions caused by *A. chabaudi* in wildcats is an interstitial granulomatous pneumonia with severe, multifocal, or coalescing damage which can be life-threatening [48,55]. There is only one description of a live wildcat affected by *A. chabaudi* but in that case clinical and radiographic manifestations of feline angiostrongylosis were not assessed, because the animal was simultaneously parasitized by other respiratory nematodes which confounded the whole scenario [56].

### 3.2. Epidemiology

In the last decade *A. chabaudi* has been found in wildcats from Germany, Greece, Romania, Bulgaria, and Bosnia and Herzegovina, and in domestic cats from Italy. These records undoubtedly prove that felids in Europe may be infected by *A. chabaudi* but, once again, retrospective epidemiological, serological, and molecular studies strongly suggest that the importance of angiostrongylosis in domestic cats is, to date, negligible [50]. Given that *A. chabaudi* shares biological, geographical, and ecological niches with other felid parasites affecting domestic cats it would have been reasonable to expect that spill-over infections could occur from *F. silvestris* to domestic cats with a significant epidemiological and clinical impact [50,57]. Apparently, this is not the case, probably because this nematode is unable to reach adulthood and sexual maturity in domestic felids for a range of immunological and anatomical reasons [54]. The reason why isolated cases of immature *A. chabaudi* in domestic cats have been recorded only in Italy could be the result of an intense parasitological pressure in given territories, and/or of greater epidemiological vigilance. Angiostrongylosis in domestic cats is not patent and the infection can be diagnosed only post mortem or with specific serological tests, thus undiagnosed cases in domestic cats from other countries cannot be excluded. *Angiostrongylus chabaudi* has never been reported in felids from America, Oceania or Asia, though findings of unspecified metastrongyloid larvae in wild felids from different countries call for further investigations [50].

## 4. *Angiostrongylus daskalovi* (Janchev & Genov, 1988)

### 4.1. Life Cycle and Pathogenesis

The DH of *A. daskalovi* is the European badger, although it has also been found in other native mustelids in Europe [8] as well as invasive American mink [58]. Reports of infection are few, but this could be confounded by uncertainty over the identification of metastrongyloid larvae in badger faeces, especially in the pre-genomic era [59]. Thus, *Aelurostrongylus falciformis* (syn. *Perostrongylus falciformis*) is a common lungworm in badgers, and infections of *A. daskalovi* might have been mistaken for this species in the past, as well as for *A. vasorum* [59,60]. Adult worms are found in the right side of the heart and pulmonary vessels, and infection is associated with pneumonia [61].

### 4.2. Epidemiology

In the single detailed study of *A. daskalovi* in badger populations, in Hungary, burdens were higher in grassland than in mixed pasture-forest or wetlands [8], perhaps due to higher badger density and/or more favourable conditions for gastropod IH, species of which have not been confirmed. The parasite has been recorded from Southern and Eastern Europe [8], although it might be under-reported elsewhere. Although prevalence can be high, sample sizes in previous studies are low, and without repeated studies, no inference can be made on whether this species is stable in distribution or expanding.

## 5. *Angiostrongylus cantonensis* (Chen, 1935)

### 5.1. Life Cycle and Pathogenesis

*Angiostrongylus cantonensis* exhibits one of the most complex circulations in ecosystems described among metazoan parasites and some of the aspects of its life cycle are also reflected in *A. vasorum*. Around the world, the natural DHs of *A. cantonensis* are several species of the genus *Rattus*, with clear dominance of the three most widely distributed invasive species: *Rattus rattus*, *Rattus norvegicus* and *Rattus exulans*. However, other rodent species can also occasionally serve as DHs, as a result of spill-over from original *Rattus* hosts [62]. The parasite also exploits an unusually broad range of gastropods as IHs [4]. In many areas of its emergence, invasive snails and slugs (i.e., *Achatina*/*Lissachatina*, *Pomacea*, *Parmarion* and *Veronicella* spp.) are considered as a major component in the epidemiology of the disease; however, local snails and slugs also serve as important hosts, for example, *Plutonia lamarckii* in the Canary Islands [63]. Importantly, both terrestrial and aquatic gastropods are involved in the life cycle and have been demonstrated as a source of human infections.

An important part of the circulation of *A. cantonensis* is also an unusually broad range of paratenic hosts that are infected by consumption of molluscs with L3 larvae or by L3 larvae liberated from their mollusc hosts. These include fish, amphibians and reptiles [64], as well as invertebrates including planarians, crustaceans, insects and centipedes [65]. L3 larvae liberated from dead or living gastropods can survive outside the IH for a month, forming an important source of infection [66]. Interestingly, the L3 larvae can enter new IHs through the process of intermediasis, which can further extend their survival [67].

A most peculiar feature of *A. cantonensis* infection is the strong neurotropism of L3 larvae in warm-blooded hosts. Almost immediately after infection, the larvae migrate into the central nervous system (CNS), where they further develop, reaching the L5 larval stage in the subarachnoidal space within two weeks post-infection [68]. This part of the life cycle usually does not produce severe signs in rats; however, infection in accidental hosts commonly results in eosinophilic meningitis with severe clinical scenarios. Clinical signs observed in infected animals result from increased intracranial pressure, neural tissue damage and host inflammatory response [69,70]. In areas of endemic occurrence, clinical cases are known among dogs, marsupials, zoo primates and in some species of bird [4,71]. In the case of the Canary Islands, dogs were found to be accidental hosts [72]. The number of accidental host species is growing, with hedgehogs recently added to the list [10]. North African hedgehogs and dogs are so far the only reported accidental hosts in Europe.

In humans, *A. cantonensis* has been reported to be the most common parasite causing eosinophilic meningitis in the tropics [2], and AEM is considered a prominent example of an emerging infectious disease. Clinical manifestations in humans are often severe, although there is a wide variety of symptoms. Long-term sequelae have been recorded, including blindness and limb paralysis [2]. Mortality is higher in children, with a case fatality rate of up to 10% [73].

### 5.2. Epidemiology

#### 5.2.1. Spread to Europe

Ongoing spread of the major DHs and IHs of *A. cantonensis* has led to an almost-global distribution of AEM within the tropics [2]. The majority of clinical cases of AEM are reported from South-East Asia; however, the disease is also prominent in Pacific regions (French Polynesia, Hawai’i, and Australia) with recent invasions into the continental United States [3,74]. Closer to Europe, *A. cantonensis* had already been detected in Egypt by the late 1970s, representing the first reports on its occurrence in the broader Mediterranean basin [75]. The intensive marine traffic through the Suez Canal might have contributed to the invasion, because the parasite chiefly spreads with its rodent DH, synanthropic rats.

It is notable that the current European foci of *A. cantonensis* are islands [9,10]. Islands are renowned for having highly endemic and disharmonic biotas in which invasive species, including introduced rodents, often play unexpected ecological roles [76]. Considering European territory, very few Mediterranean islands remain rat-free: 99% of islands in the western basin have been invaded by rats [77]. The presence of *A. cantonensis* in *R. rattus* from the island of Tenerife, Canary Islands, was recorded in 2010 [9], but the islands are distant from mainland Europe, and this can be considered an extension of the African range. In this Atlantic archipelago, the two rat species present, *R. rattus* and *R. norvegicus*, act as DHs for *A. cantonensis* [78], and the mollusc species *P. lamarckii*, *Cornu aspersum* and *Theba pisana* were identified as IHs [63]. A survey carried out in the eight islands of the archipelago demonstrated the presence of *A. cantonensis* only in Tenerife [78], one of the two most populated islands. The Canary Islands play an important role as a maritime connection between European, African, and American ports, that could imply a risk of spread of *A. cantonensis* to new locations.

The parasite was further detected in 2018 in North African hedgehogs (*Atelerix algirus*) from Mallorca [10]. Mallorca is the largest island of the Balearic Archipelago, located in the Mediterranean Basin. This unexpected discovery was the result of passive parasitological surveillance in local wildlife. Since its first detection, the parasite has been found repeatedly in several foci in the island, particularly during autumn and winter seasons (Paredes-Esquivel, unpublished). Surveillance has been restricted to hedgehogs, due to resource limitations, and this species is currently being used as a biosentinel. Considering the large home range of these mammals, the exact locations where the parasite is currently circulating in Mallorca are still unknown. There is no information either on the DH or IH species involved in transmission. There is an urgent need for further studies and surveillance in local hosts to untangle the epidemiological drivers of the disease in the Balearics, especially because these are likely to inform probable conditions for establishment in neighbouring areas.

#### 5.2.2. Risk Factors for Disease

The complex life cycle, discrete localization in infected animals and humans (circulatory system, brain) and presence in a wide range of intermediate and paratenic hosts (molluscs, amphibians, fish, reptiles, crustaceans) represent obstacles for diagnosis of AEM. Human infection occurs via ingestion of third stage L3 larvae, most commonly by eating an infected raw snail, deliberately or inadvertently—commonly on contaminated salad or vegetables—or by the consumption of infected poikilothermic hosts (fish, frogs, reptiles, and crustaceans) [2]. The same routes of infection can be anticipated in other accidental mammalian hosts.

Thus far, almost all cases of AEM in Europe have been in patients travelling from known endemic areas in the tropics [79], most of whom were diagnosed following the investigation of persistent headaches. One case, however, occurred in a woman in Paris, France, with no history of recent travel outside the country or eating imported high-risk foods [80]. It is therefore possible that undiagnosed endemic AEM cases are already occurring in Europe, and that *A. cantonensis* is more widely distributed than is known.

Clearly, the confirmed presence of *A. cantonensis* on European islands poses a significant zoonotic risk, and although AEM cases local to the reported incursions have not yet been reported, vigilance is required. In the Canary Islands, local research projects have included the analyses of human samples of the main public hospitals of Tenerife, and, up to now, one positive case has been observed [63]. At present, these analyses are still being carried out in order to detect more possible cases of angiostrongyliasis in these islands. The early detection of *A. cantonensis* in Mallorca has also enabled the inclusion of this parasite in the differential diagnosis of AEM in local hospitals [81]. Going further, future cases of AEM anywhere in Europe should trigger local epidemiological surveillance if travel-related risk factors are absent.

The main risk factors for human infection are food-borne, and food safety in endemic areas should be re-considered to include AEM risk management. In the Mediterranean region, including the Balearics, snails are an important part of the local cuisine. Infections by eating snails are unlikely because these are thoroughly cooked. However, social alarm may negatively affect the local heliciculture industry if products are perceived as unsafe. Furthermore, it is notable that the dietary risk factor identified in imported human cases was more often freshwater shrimp or salad than snails. The Mediterranean and Canary diet also includes a high intake of raw vegetables, particularly salads. Thus, although raw snails and slugs are not part of the diet, there is a risk associated with the ingestion of undercooked *C. aspersum* that is cooked for food, or accidentally ingesting snails or slugs in vegetable produce [63].

With the parasite actively circulating in the archipelagos, there is an urgent need to establish food monitoring and surveillance strategies to prevent the risk of infection. Prevention of food contamination by gastropods, the cooking or freezing of known IHs, and paratenic hosts before consumption, and surveillance and food inspection in high-risk areas are recommended. This is perhaps especially pertinent because arthropods, and invertebrates in general, are promoted as part of sustainable future diets [82]. Traditional medicinal consumption of raw gastropods also occurs in the Mediterranean region and has been implicated in cases of toxocariasis [83].

#### 5.2.3. Chance of Further Spread

The epidemiology of AEM involves humans, poikilothermic and homoeothermic vertebrates, and invertebrates, as well as environmental components; therefore, the One Health approach is highly appropriate to seek holistic understanding of its transmission and disease. In invaded ecosystems, rats and snails are the core source of data for distribution mapping, although accidental and paratenic hosts can also serve as sentinels for *A. cantonensis* detection. Demonstrations of the common occurrence of *A. cantonensis* infections in reptiles [84,85] or hedgehogs [10] serve as a proof of concept of such an approach. In Australia, disease in domestic dogs [86] and in birds [87] reflected southward expansion of *A. cantonensis*, and the monitoring of animal disease has an important role to play in the epidemiological surveillance of AEM risk in Europe.

In terms of the risks of *A. cantonensis* spread within Europe, similar ecological conditions to those on Mallorca exist on the 190 islands located in the Mediterranean Basin, as well as adjacent mainland. Risk of spread to these areas is elevated by the strong maritime connections and high movement of tourists between the islands and continental Europe.

In the absence of solid understanding of the ecological and climatic factors underlying suitability for the establishment of *A. cantonensis* in Europe, and the behavioural and dietary factors locally important to human exposure, the future trajectory of AEM on the continent, and potential bioclimatic limits to its eventual range, are difficult to predict. Climate and environment are certainly important to parasite distribution, including on Tenerife [78], and environmental correlates of existing distribution have been used elsewhere, alongside climate change projections, to predict areas at risk of future invasion [88]. Globally, similar models have suggested a decrease in the total habitat suitable for *A. cantonensis* under climate change [89], but in spite of this, continued spread and introduction events potentially increase actual range [90]. Correlations between environmental variables and observed parasite distribution or AEM occurrence in places such as South-East Asia or Hawai’i, however, might not predict the conditions leading to high or low transmission probability in Europe. Further epidemiological research in the new foci of Europe would seem critical to understanding the prospects of future spread on the continent and strategies for its prevention.

## 6. *Angiostrongylus dujardini* (Drozdz & Doby, 1970)

### 6.1. Life Cycle and Pathogenesis

Rodents act as DHs: most notably in Europe are the wood mouse *Apodemus sylvaticus* [91] and bank vole *Myodes* (syn. *Clethrionomys*) *glareolus*. In common with other *Angiostrongylus* species, the worms reside in the heart and pulmonary vessels, and use gastropod IHs. It is in accidental hosts that disease is most noteworthy, however, because larvae migrate within them and cause inflammatory lesions. Outbreaks in zoological collections have been reported, involving callitrichid monkeys (tamarins and marmosets) and meerkats. Generalized migration of larvae was observed to tissues including the lungs, liver, heart, kidneys and intestinal wall, and signs varied from sudden death to general malaise, anorexia and dyspnoea [11,92]. In some cases, adult worms were recovered from the heart, and lesions in some tissues surrounded eggs and larvae that must have been shed from mature adults; however, no larvae were identified in the faeces.

### 6.2. Epidemiology

Little is known about the epidemiology of this species. In wood mice in Portugal, prevalence was higher in adults and in more open habitats [91], indicating that ecological factors are likely to be important in parasite maintenance. The reported outbreaks in zoological gardens were also in Southern Europe, and presumably arose from the invasion of enclosures by infected rodent and/or gastropod hosts. Infected *Apodemus* mice were found in one zoo [92], and in the other the outbreak followed an unusually rainy period during which gastropods were noted to be abundant [11]. Diagnosis is almost impossible in living animals, and even post-mortem requires awareness of the possibility of *A. dujardini* infection; therefore, it is probable that the distribution of the parasite and associated disease are underestimated, whereas this lack of information precludes the assessment of possible range expansion and future threats.

## 7. Gaps in the Understanding Needed for Risk Prediction and Management

The epidemiology of *Angiostrongylus* spp. infection is extremely complex. Ecological, behavioural and genetic factors increasingly seem important to the geographic range and disease impact of species in the genus, but their study is in its infancy and firm conclusions are elusive. The remainder of this review seeks to identify the most important gaps in understanding, which must be addressed in order to properly assess the risk presented by *Angiostrongylus* spp. in Europe.

### 7.1. Ecology

As research discovers new hosts for different *Angiostrongylus* spp., their contribution to parasite maintenance is questioned, but generally remains unconfirmed. Logically, the most abundant and commonly infected natural DH and IH species are likely to drive transmission, such as rat DHs for *A. cantonensis* and fox DHs for *A. vasorum*; however, other rodents and carnivores, including domestic dogs for *A. vasorum*, might be important in local transmission cycles. For IH, as far as is known all *Angiostrongylus* species are indiscriminate in their ability to develop different gastropod species, but variation in the larval yield of artificial infections, perhaps mediated by defence mechanisms [93], and highly variable prevalence in natural infections, suggest that IH availability could act as an ecological filter. Spatio-temporal variation in the species composition of gastropod communities, and its environmental correlates, could therefore influence *Angiostrongylus* spp. maintenance and emergence in different areas. Ecological network approaches, including trophic relationships between hosts, could shed further light on the conditions for parasite persistence and the risk of spill-over to domestic animals and humans. The impacts of infection on natural populations also remain unknown, and pathological manifestations in infected animals suggest that fitness could be affected in DHs, IHs, paratenic and accidental hosts. Invasion of rats and the urbanization of foxes might well accelerate the emergence of *Angiostrongylus* spp., but additional ecological processes are involved and must be considered in aggregate if holistic understanding is to be achieved.

### 7.2. Behaviour

Behaviour, especially in relation to feeding and habitat use, is likely to strongly affect the transmission of *Angiostrongylus* spp. and consequent parasite maintenance, emergence and disease risk. Notwithstanding the possibility of infection of DHs and accidental hosts with L3 larvae living free in the environment following emergence from infected IH, most links between IHs and DHs in both directions, and involvement of other hosts in the life cycle, are mediated by trophic interactions. L1 larvae are acquired by gastropod IH feeding on DH faeces, and this might account for the relatively high prevalence of *A. vasorum*, for example, in coprophilic gastropod species. Within-species variation in the diet of *Angiostrongylus* spp. DHs might account for spatial and demographic variations in the prevalence of infection, as observed for *A. dujardini* [11] and *A. daskalovi* [8]. Ingestion of gastropod IH is also implicated in the infection of DH and accidental hosts in zoological collections. For humans, eating gastropods or known paratenic hosts, accidentally or intentionally, is a prominent risk factor for AEM. It is tempting to attribute the limited evidence of autochtonous AEM in Europe despite the local presence of *A. cantonensis* to dietary behaviours that either avoid IH and paratenic hosts altogether, or cook them before consumption. Other behaviours are relevant to the transmission of *Angiostrongylus* spp., including the walking of dogs in areas with a greater or lesser availability of gastropod IH, and by implication, a risk of *A. vasorum* [94]. The role of behaviour in *Angiostrongylus* spp. transmission has been little studied in any part of the life cycle and could be important to parasite maintenance and to strategies of reducing infection risk, including for AEM.

### 7.3. Genetics

There is abundant evidence for genetic diversity within *Angiostrongylus* species, and this has been proposed to be linked to pathogenesis and epidemiology, e.g., *A. vasorum* [95,96,97]. As a result, extrapolation between studies should be tentative when different parasite strains might be involved. For example, European populations of *A. vasorum* are genetically very different from those in Brazil [98], and it should not be assumed that biological or pathological characteristics of the two populations are the same. In *A. cantonensis*, certain genotypes dominate in invaded islands, and genetic proximity between distant insular populations suggests that beyond possible founder effects, there are parasite strains that are invasive by nature [99]. The mitochondrial haplotypes (using the Cytochrome Oxidase C I gene region) present in Mallorca and Tenerife are the same (Paredes-Esquivel, unpublished). However, given the recent expansion of *A. cantonensis*, this marker may not be informative enough to trace the invasion process.

Host genetic variation might also influence epidemiology and disease emergence; for example, it is notable that haemorrhagic disease is not elicited in artificial infections of *A. vasorum* in inbred dogs or in foxes, but occurs in a subset of naturally infected dogs, although explanations other than host genetics are possible. Associations between genotype and the distribution, transmission and outcome of *Angiostrongylus* spp. infections have therefore been demonstrated, but the causation and mechanism have not been established. Patterns of genetic diversity can be informative in tracing past parasite invasions and inferring biogeography in historic and prehistoric timescales, e.g., the invasion of North American islands with European *A. vasorum* [98]. The arrival of *A. cantonensis* in Europe in the genomic era provides an opportunity to document and track parasite genetics from a foundation event, and—should further spread occur—provide insights into its mechanisms and strategies for control.

### 7.4. Climate

Climate determines the development rate of *Angiostrongylus* spp. in IH, and the abundance of IH and their availability to other hosts in the life cycle. The current tropical distribution of *A. cantonensis* provides reassurance that it is unlikely to spread to the north of Europe, whereas *A. vasorum* appears to be excluded from very cold or very dry regions [40]. Empirical approaches that correlate climate with parasite occurrence, however, have limitations when evaluating potential parasite spread into new areas or with climate change, where relationships between climatic drivers and the occurrence of the parasite or disease are modified by complex factors that vary between regions and potentially over time. Predictions based mainly on observed distribution, i.e., the realized range [40,88,89] might consequently be limited for complex and heterogeneous parasite systems, especially when dynamics are unstable. Gastropod abundance and activity, for example, are strongly affected by humidity, not only temperature, complicating climate-based projections, although based on current understanding it is hard to see how the multiple and context-specific ecological factors important to *Angiostrongylus* spp. can be robustly incorporated into life cycle models. Modelling of other infectious diseases has involved sustained efforts over many years, although all started with simple approaches, and augmenting this effort for *Angiostrongylus* spp. could reap dividends for understanding and risk management.

### 7.5. Surveillance

In the absence of systematic compilations of animal disease occurrence, especially in wild and non-food domestic animals, few tools exist for the passive surveillance of *Angiostrongylus* spp. Prospective studies, for example through parasitological or serological sampling of DH and IH, have been important to current understandings of *A. vasorum* range and ecology. Although these studies were not systematic, they exceed what is currently available for other species. Reporting of confirmed cases of AEM is likely to be better organized. However, the specific diagnosis of diseases is difficult, because recognition of those caused by *Angiostrongylus* spp. rely heavily on clinical awareness, which can lag considerably behind parasite spread. This limitation, along with lack of mechanisms for surveillance, led to substantial and avoidable misdiagnosis of canine angiostrongylosis during its expansion across Europe. Awareness of AEM among medical professionals, including thorough patient histories, and complementary surveillance in animals, will be important to recognize disease and to properly evaluate geographical spread and risks of infection.

The detection of *A. cantonensis* in wildlife in Europe [10] and elsewhere [87], along with numerous surveys of *A. vasorum* in wild carnivores [31,32,33,34], point to the value of wildlife surveillance in detecting range expansion. Animals in zoological collections should be included in this, especially for *A. dujardini* [11,87] and also *A. vasorum* [29,100,101,102].

## 8. Concluding Remarks

**Figure 1:**
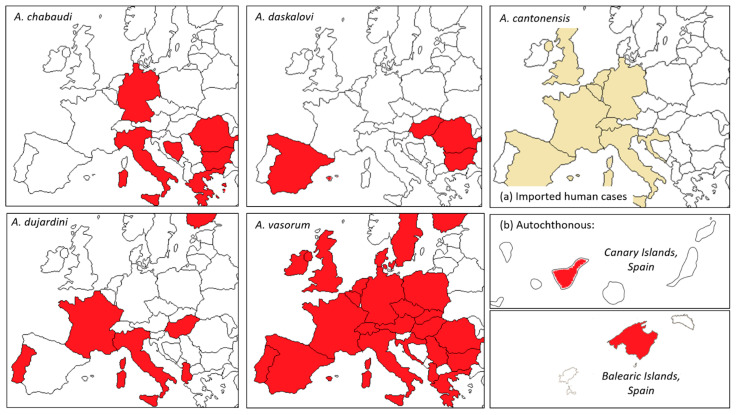
Recorded ranges of *Angiostrongylus* spp. in Europe. Maps show the location of reports by country (and, for *A. cantonensis*, by island), and not distribution within countries or islands. Sources: *A. cantonensis*: Imported human cases (**a**); Autochthonous (**b**) [10,78], *A. chabaudi* [47,48,49], *A. daskalovi* [8,58,59,60,61], *A. dujardini* [11,91,92], *A. vasorum* [13,30,31,32,33,34,35,36,37].

The emergence of *A. vasorum* in Europe, and the recent arrival of *A. cantonensis*, have focused attention on this complex but neglected genus, and highlighted the presence and limited knowledge of other species within it, namely, *A. chabaudi*, *A. daskalovi* and *A. dujardini* (Figure 1). The ecology of *Angiostrongylus* spp. is extremely complex, involving myriad core (DH, IH) and auxiliary (paratenic, accidental) hosts. Relationships between hosts govern parasite maintenance and emergence into domestic animals and humans, and are, in turn, influenced by environment, climate and behaviour. Greater understanding of the biology, ecology and epidemiology of this genus is needed if the prospects of future spread and disease in Europe are to be evaluated and managed.

## Figures and Tables

**Table 1:** Hypothetical factors underlying spread of *Angiostrongylus vasorum* in Europe.

Factor	Evidence for	Evidence Against
Awareness of veterinary clinicians	↑ Vigilance and new diagnostic tests lead to more records and apparent spread [30]	Expansion and increased prevalence in foxes over time against robust baseline, not affected by clinical awareness [31,32,33,34]
Fox urbanization	Emergence in Western Europe followed rabies eradication and ↑ urban foxes, c.f. *Echinococcus multilocularis* [41]	In U.K. ‘hotspots’, urban foxes were common long before emergence, which also coincided with relatively low populations due to scabies outbreaks [42]
Gastropod invasion	Emergence along with the invasion of *Arion vulgaris* (syn. *lusitanicus*), e.g., in Denmark [43] and Switzerland [44]	Infects a very broad gastropod range, including displaced species; emergence in areas without much *A. lusitanicus* invasion, e.g., United Kingdom
Climate change	Predicted increased development rate in IH with temperature, based on other metastrongyloids; climate change can favour gastropod abundance [45,46]	Newly colonised areas are predicted to be historically suitable for the parasite [40]; climate change can inhibit as well as improve conditions for gastropods [45]
Dog movement	Longer-range dispersal ahead of spread in foxes, e.g., in the United Kingdom [16]	New records in areas long subject to dog movements [33,34,35,36,37]
